# Knowledge on Signs and Risk Factors in Stroke Patients

**DOI:** 10.3390/jcm9082557

**Published:** 2020-08-07

**Authors:** Raúl Soto-Cámara, Jerónimo J. González-Bernal, Josefa González-Santos, José M. Aguilar-Parra, Rubén Trigueros, Remedios López-Liria

**Affiliations:** 1 Department of Health Sciences, University of Burgos, 09001 Burgos, Spain; rscamara@ubu.es; 2 Department of Psychology, Health Research Centre, University of Almeria, 04120 Almeria, Spain; jmaguilar@ual.es; 3 Department of Nursing, Physiotherapy and Medicine, Health Research Centre, University of Almería, 04120 Almeria, Spain; rll040@ual.es

**Keywords:** stroke, risk factor, knowledge, signs, health education

## Abstract

Background: There is a pressing need to contribute evidence to the improvement in the early identification of signs and symptoms associated with strokes, and address the treatment-seeking delays. The objective of this study is to describe the knowledge regarding the warning signs and risk factors (RFs) among stroke patients, as well as of their attitudes toward a suspected event, and the analysis of its possible relationship with the socio-demographic and clinical characteristics of these patients. Method: A cross-sectional study was designed, in which all stroke patients admitted consecutively to the Burgos University Hospital (Spain) were included. The principal outcomes were the patient’s ability to identify two RFs and two warning signs and the patient’s hypothetical response to a possible stroke event. The possible factors associated with the knowledge of warning signs, RFs, and the correct response to a new event were studied using univariate and multivariate regression analysis. Results: A total of 529 patients were included. Having a higher education level or a history of prior stroke were associated with a greater degree of knowledge of warning signs (odds ratio (OR) 3.19, 95% confidence interval (CI) 1.70–5.74, *p* = 0.003; OR 3.54, 95%CI 2.09–5.99, *p* ≤ 0.001, respectively), RFs (OR 3.15, 95%CI 1.75–5.67, *p* = 0.008; OR 4.08, 95%CI 2.41–6.91, *p* = 0.002, respectively), and the correct response to a possible stroke (OR 1.82, 95%CI 1.16–2.86; *p* = 0.030; OR 2.11, 95%CI 1.29–3.46, *p* = 0.022, respectively). Conclusion: Knowledge of warning signs or stroke RFs is low in the hospitalized patients. A previous stroke or secondary/higher education levels are the predictor factors that increase the probability of knowledge of warning signs, RFs, or reaction to possible event.

## 1. Introduction

Strokes continue to be a global health issue and a social problem, a situation expected to worsen in the coming years as the population ages. Strokes represent one of the major causes of mortality, dependency, and long-term disability in adults, proving to be highly expensive for the patients, their families, the health services, and the community as a whole [1,2].

The treatment of strokes is a dynamic process, in which time is the most critical factor impacting the appropriate performance of the interventions held for acute stroke and determining the patient’s final prognosis [3]. Many patients do not receive treatment because of the delay in the presentation to the hospital, which results in exceeding the time point at which the treatment is efficacious. Several studies have identified that one of the main reasons behind the extension of this timeframe, between the onset of the symptoms and the arrival at the hospital, is the lack of knowledge regarding the warning signs and risk factors (RFs) associated with stroke [4,5,6,7,8] among the patients or witnesses, which in practice translates into refusal on the part of the patients to acknowledge their disease [9,10], or a hope that the symptoms would fade or improve on their own [11,12]. In addition, despite the high prevalence and severity of stroke, the knowledge regarding the disease in the general population is lower compared to the other diseases, such as acute coronary syndrome (ACS), cancer, or acquired immunodeficiency syndrome (AIDS), even in the patients who have already experienced a stroke [13]. Public awareness messages, such as “Stroke Chain of Survival” [14], “Time is Brain” [15,16], or “Face, Arms, Speech, Time (FAST)” [17], have been spread and programs have been developed in order to accentuate stroke as a condition of medical emergency and to reduce delays in hospitalization. These programs are based on the premise that better knowledge of the warning signs associated with stroke would lead to improved recognition of the possible event and an immediate call and activation of the pre-hospital emergency team [18]. Knowledge regarding the RFs associated with stroke might improve the primary and secondary prevention, and encourage people to adopt preventive behaviors through lifestyle modification, leading to a decreased incidence of cerebrovascular problems in the future [18]. Previous studies have demonstrated that a significant proportion of patients who are at a high risk of stroke are unaware of this risk [6,19,20].

Most of the previous studies have aimed at assessing community knowledge regarding the stroke-associated RFs, warning signs, and the attitudes intended to be undertaken when such an event is suspected [21,22,23,24]. Although, a smaller number of studies assessing these aspects specifically in the patients that have experienced a stroke are also available in the literature [25,26,27].

The objective of this study was, therefore, to describe the stroke patients’ knowledge regarding the warning signs and RFs associated with stroke, as well their attitudes toward a suspected event, and analyzing the possible relationship of this knowledge to the socio-demographic and clinical characteristics of the patients.

## 2. Materials and Methods

### 2.1. Design—Study Population

This is a cross-sectional prospective descriptive study, part of a larger project conducted on pre-hospital delay and stroke knowledge and treatment [28,29]. The STROBE statement of this cross-sectional study is in the Supplementary Files. The study population was a consecutive cohort of patients aged 18 years or above, admitted with an initial probable diagnosis of stroke in the Neurological Department of Burgos University Hospital (Spain), during the period of over 12 months. The World Health Organization has defined stroke as the “rapidly developing clinical signs of focal (at times global) disturbance in cerebral function, lasting for more than 24 h or even leading to death, with no apparent cause other than a vascular origin” [30]. Stroke was diagnosed and confirmed by computed tomography and/or magnetic resonance imaging of the brain, performed at the acute stage in all patients [14]. The cases of in-hospital stroke (defined “as acute infarction of central nervous system tissue that occurs during hospitalization in a patient originally admitted for another diagnosis or procedure”) [31], as well as the patients for whom it was not possible to perform anamnesis due to prior cognitive impairment or alteration in the level of consciousness or severe aphasia, the patients who were discharged within the first hour after admission, and those who expressed refusal to participate in the study, were excluded from the study. If during study duration (one year), a patient was admitted to hospital due to stroke on more than one occasion, only the first episode was considered for data collection. No mass-media public stroke-awareness campaign was conducted over the course of the study. All patients were managed according to the standard stroke guidelines [14].

### 2.2. Procedure—Data Collection

All the patients were invited to participate in the study on a voluntary basis, ensuring that their identity would remain anonymous. The patients and/or their relatives were informed of the nature of the research and its objectives and were asked to sign a consent form if they agreed with it. The patients were allowed to withdraw at any point of time. Data were collected by conducting structured clinical face-to-face interviews by a member of the research team in the first 48 h post-admission, with the aim of minimizing the effect of the hospital stay on the research factors, after the patient’s electronic medical records were reviewed. Both the patients and their relatives could answer questions about socio-demographic and clinical characteristics, whereas stroke-related knowledge had to be answered by the patients themselves. The time taken by each participant for completing the interview varied from 10 to 15 min.

Owing to the non-existence of the Spanish validated tools, a questionnaire was designed ad hoc specifically, based on those used in similar previous studies [32,33,34]. The questionnaire was pilot-tested on 25 patients who were not a part of the study population, prior to being used for analysis in this study, in order to ensure that the questions were clear and understandable for the reader. No changes in the wording of the questions were performed as a result of the pilot study. The final questionnaire included questions related to the socio-demographic and clinical characteristics of the patients and their stroke-related knowledge. The last part of the questionnaire had three questions related to stroke-associated symptoms, RFs, and the attitudes toward a possible event. Internal consistency of the sets of questions was assessed by means of Cronbach’s alpha factor, the value of which was determined to be satisfactory (0.87). The research protocol was approved by the Institutional Review Board (IRB–1479–2015).

### 2.3. Main Outcomes

The primary outcome was the patients’ previous knowledge regarding stroke, which was assessed through their ability to identify at least two RFs and two warning signs associated with stroke, as well as by their hypothetical responses to a possible stroke in the event of witnessing a stroke event or experiencing one.

In order to assess the first two aspects, the following two open-ended questions were asked: “What stroke warning signs are you aware of?” and “What stroke RFs are you aware of?” According to the National Institute of Neurological Disorders and Stroke (NINDS) classification [35] and the Spanish Neurological Society (Sociedad Española de Neurología, SEN) [36], valid warning signs of a stroke were as follows: loss of strength or sudden weakness in the face, arm, or leg, especially on one side of the body; a sensitivity disorder in the face, arm, or leg; sudden confusion or difficulty in speaking or understanding language, a sudden change in one’s sight in one or both the eyes; sudden problems in walking; dizziness and/or loss of balance or coordination; and a sudden, intense headache without a known cause. The stroke RFs considered to be possible correct answers were [37,38,39,40]: old age, a previous stroke, high blood pressure (HBP), diabetes mellitus (DM), dyslipidemia, overweight or abdominal obesity, cardiovascular disease, active smoking habit, excessive alcohol intake, diet risk score, a sedentary lifestyle, and psychosocial factors. Only the first two symptoms or RFs mentioned by the patients were considered as responses.

In order to assess a person’s attitude to a possible stroke event, the following closed-ended question was asked: “If you think someone is having the symptoms of a stroke, which will be your first reaction?” The three options to choose from as a response to this question were as follows: (1) No specific action is required; (2) Treatment should be sought, although not necessarily immediately; and (3) This is an emergency and medical attention should be sought immediately; the correct one being the one which recognized the possibility of treatment and the importance of the time factor.

The patients who were able to correctly answer at least one question were then asked how they acquired this knowledge.

Socio-demographic information such as age, sex, civil status (married/living with a partner, single/widower), maximum educational level (no studies/primary, secondary/higher education), employment status (employed, unemployed), average yearly income (<EUR 20,000, ≥EUR 20.000), living situation (living in a household accompanied by at least two residents, living alone), and the area of residence (urban, rural), as well as clinical data (past medical history and stroke signs and symptoms) were collected. For the evaluation of the stroke RFs were used the criteria defined by the INTERSTROKE project [37,38] and the Program of Preventive Activities and Health Promotion of the Spanish Society of Family and Community Medicine (PAPPS-SEMFYC) [39,40]. Operative details and outcomes have been reported previously [28,29].

### 2.4. Statistical Analysis

A descriptive analysis of the variables was undertaken. The categorical variables were expressed as absolute frequencies and percentages, while the continuous variables were expressed in terms of mean and standard deviation (SD). A chi-squared test was used to assess the possible differences in the categorical variables based on prior stroke knowledge, and the characteristics of both the groups were compared by using Student’s *t*-test for independent samples in the event of them being continuous. Forward stepwise multivariate regression analysis adjusted by age and sex was conducted in order to identify the possible factors associated with sufficient stroke knowledge, which included the variables of statistical significance identified in the univariate analysis. The entry criteria for the multivariate model was a *p*-value of ≤0.05 in the previous univariate analysis. As a measure of association, odds ratio (OR) was used with a 95% confidence interval (CI). A *p*-value ≤0.05 were considered statistically significant. SPSS version 25.0 (IBM SPSS Inc, Chicago, IL, USA) was utilized for performing the data analysis.

## 3. Results

### 3.1. Characteristics of Participants

Among the 583 eligible patients, 54 were excluded because of different reasons: in-hospital stroke cases (*n* = 21), recurrent stroke during this study duration (*n* = 19), the impossibility of follow-up (*n* = 8), and inability to communicate data (*n* = 6). The characteristics of the excluded group of patients, with respect to age and gender, were similar to those of the 529 patients included in this study.

The socio-demographic and clinical profiles of the participants are summarized in Table 1. The study sample comprised 529 patients, with a narrow majority of men over women (55.77% versus 44.23%). The age of the participants ranged from 25 to 99, with an average age of 75.39 (SD ± 12.67). Among the participants, 84.31% had experienced an ischemic stroke, while 15.69% had experienced a cerebral hemorrhage. High blood pressure (67.49%) and overweight/obesity (64.84%) were the most frequent RFs reported by the patients, while the loss of strength/weakness (66.73%) and speech/language disturbance (58.98%) were the most common symptoms that were reported.

### 3.2. Knowledge of Warning Signs

Among the 529 patients, 299 (56.52%) identified two warning signs, with the loss of strength (30.25%) and sensitivity (22.87%) as the most frequently reported ones. When the recognition of the warning signs from FAST [17], a scale with a high predictive value which is used at the pre-hospital level to facilitate the identification of a stroke patient, was assessed it was observed that only 18 patients (3.40%) were able to recognize them.

The proportion of patients who, having experienced a certain warning sign during the acute stroke phase, were able to identify it as a characteristic of a cerebrovascular event, ranged between 1% and 20%, with lower percentages mentioning alteration of vision or speech/language disturbance. None reached the threshold levels of statistical significance when compared to the percentage of patients who could not identify these characteristics (Table 2).

Lower age (*p* ≤ 0.001), being male (*p* = 0.002), being married or living with a partner (*p* = 0.037), having a secondary/higher level of educational attainment (*p* ≤ 0.001), being in employment (*p* = 0.014), having an annual income of over EUR 20,000 (*p* = 0.002), having experienced a stroke previously (*p* ≤ 0.001), no personal history of HBP (*p* = 0.021), and not leading a sedentary lifestyle (*p* = 0.009) were observed to be individually related to the correct identification of the warning signs of stroke. After the multivariate analysis, the probability of the knowledge of warning signs of stroke was four times greater in the patients who had previously experienced a stroke (OR 4.08, 95%CI 2.41–6.91; *p* = 0.002) and three times greater in the patients with a secondary/higher level of educational attainment (OR 3.15, 95%CI 1.75–5.67; *p* = 0.008) (Table 3).

### 3.3. Knowledge of RFs

Among the participants, 53.50% (*n* = 283) identified two RFs, being cardiovascular disease and high blood pressure the most frequently cited, in the 22.87% and 22.68% of the cases, respectively; while no patient mentioned having an unhealthy diet or psychosocial factors. Active smoking and high blood pressure were the most prevalent modifiable RFs in stroke patients [37,38], both affecting 13 (2.46%) patients in this study.

The proportion of patients who identified a certain RF ranged between 5% and 20%. The comparison of the patients, those who were unable to identify the RF with those who were able to, revealed statistical significance in cases where the person had previously experienced a stroke (*p* = 0.002) or was an active smoker (*p* ≤ 0.001) (Table 4).

Lower age (*p* ≤ 0.001), being male (*p* = 0.005), having a secondary/higher level of educational attainment (*p* ≤ 0.001), being in employment (*p* ≤ 0.001), having an annual income of over EUR 20,000 (*p* ≤ 0.001), having previously experienced a stroke (*p* ≤ 0.001), no personal history of high blood pressure (*p* ≤ 0.001), personal history of high alcohol consumption (*p* = 0.003), being an active smoker (*p* ≤ 0.001), and overweight/obesity (*p* = 0.005) were observed to be individually associated with the correct identification of warning signs of stroke. After the multivariate analysis, having previously experienced a stroke (OR 3.54, 95%CI 2.09–5.99; *p* ≤ 0.001) and having a secondary/higher level of educational attainment (OR 3.19, 95%CI 1.70–5.74; *p* = 0.003) were revealed as the predictors of knowledge of RFs (Table 3).

### 3.4. Reaction to a Possible Stroke Event

Among the participants, 26.46% (*n* = 140) stated that, in response to a possible stroke event, they would immediately seek assistance, as it would be a medical emergency, 31.57% (*n* = 167) stated that they would take no specific action, and 41.97% (*n* = 222) stated that they would seek assistance, although not immediately.

Having a secondary/higher level of educational attainment (*p* = 0.033) or having experienced a stroke previously (*p* ≤ 0.001) were observed to be individually associated with the seek assistance immediately in the event of a suspected stroke option, with a statistical significance that was maintained in the multivariate analysis with OR values of 1.82 (95%CI 1.16–2.86) and 2.11 (95%CI 1.29–3.46), respectively (Table 3).

When a patient’s reaction to a new event was related to their knowledge of the warning signs of a stroke, it was observed that 21.36% (*n* = 113) of the patients identified the signs correctly and related them to an emergency (*p* ≤ 0.001).

### 3.5. Source of Information

The analysis of the way in which the patients had acquired the knowledge of warning signs revealed that 40.64% (*n* = 215) of the patients had attained the knowledge through personal experience or from their immediate environment, 38.75% (*n* = 205) had attained the knowledge through the media, and 20.61% (*n* = 109) had attained it from healthcare professionals.

No statistically significant differences were observed when relating stroke knowledge to the source of the information (*p* = 0.735).

## 4. Discussion

The results of this study are similar to those obtained by the other researchers working with stroke patients [25,26,27,32,33,34] or the general public [21,22,23,24,41,42,43], demonstrating a lack of knowledge of the stroke-related warning signs, and how to act in a possible event of a stroke. In this situation, the patients might delay seeking assistance and arrive late at the hospital, which would have important repercussions in regard to their final prognosis, in terms of dependency and disability. In addition, stroke patients may have significant deficits, such as aphasia, reduced consciousness, or cognitive impairment, which prevent them from seeking assistance and ultimately increasing the pre-hospital delay [44].

There is not much uniformity in the results of the research studies in terms of the participants, the definition of knowledge and the methodology used, which creates difficulty in making comparisons. The participants may be from the general public [21,43,45,46], the patients who had experienced a stroke [25,26,27,32], or those who were at serious risk of suffering a stroke [33,47]. The type of questionnaires used in the previous studies, with questions ranging from open-ended questions [27,32,34] to multiple-choice options from with the patient has to identify and select the correct answer(s) [25,26,33,47], may exert an influence on the results, underestimating or overestimating the real level of knowledge that the patients have.

In this study, the proportion of patients who could correctly identify stroke warning signs have been slightly higher than that observed in the previous ones [26,33,48,49,50], although the lack of knowledge is nonetheless high. Loss of strength and sensitivity has been the issues mentioned most frequently, confirming in part those noted by other researchers, who conclude that loss of strength and speaking/language, both warning signs valued in the FAST scale [17], are mentioned by the majority of the patients participating in their studies [26,27,34,47,51]. According to the results of a qualitative study conducted by Sug Yoon et al. [52] in Australia, patients who survive a stroke think that their symptoms are unrelated to the information they have previously received about the disease.

In regard to the knowledge of RFs, in this study, the percentage of patients who are able to correctly name these factors are in the mean range of results of the previous ones [19,26,32,47,48,51]. High blood pressure and smoking are the two RFs most commonly identified by the other researchers [19,27,32,34,50]. However, cardiovascular disease is the most commonly mentioned factor in this study, which could be due to the attention this disease has received from the healthcare system. Meanwhile, lifestyle-related factors, such as for overweight/obesity and physical inactivity, despite their pervasiveness and increment of the stroke risk, are identified only in a limited number of cases (4.16% and 6.81%, respectively). Only a small proportion of patients are able to identify their own RFs, which may lead to the continuation of an unhealthy lifestyle or poor medical compliance. This aspect should be considered in the prevention of new episodes, since it is the previous step before a change in behavior. For example, alcohol consumption was recorded excessive if the patient drank more than 9 g of alcohol per day in men and more than 6 g in women; in this study almost 50% of patients have an excessive alcohol consumption, but only 3% recognized this risk factor as characteristic of stroke. Gupta et al. [20], in a study undertaken in the United Kingdom on patients aged 65 years with personal history of stroke, AF, HBP, or DM, have observed that 65% of them are able to mention at least one RF, with up to 85% of them not considering themselves to be in danger of having a stroke. In a study published by Kothari et al. [51], only 31% of the hypertensive patients have mentioned HBP as a stroke risk, despite suffering the condition.

In this study, one in four patients have identified stroke as an emergency, and have stated that they would seek immediate medical assistance as their first response in a hypothetical case. This result contrasts to the 89% proportion reported by Droste et al. [34]. Identifying stroke warning signs and RFs has not been associated with greater recognition of the situation as an emergency. Despite the fact that 41.97% of the participants have responded correctly to the questions related to warning signs and RFs, only 16.99% of them knew what was the correct way to react in a hypothetical situation, i.e., to call the emergency medical services or to visit the hospital directly. These findings highlight the limited capacity of the patients to automatically transform knowledge into action [53]. Seeking assistance or visiting a health center depends more on considering the severity of the symptoms rather than on one’s knowledge of the disease [54]. Although certain studies have highlighted that the lack of knowledge further increases the delay in visiting a health center [55]. Jones et al. [56], in a review study analyzing the awareness of the steps to be undertaken after a stroke event and the patient’s reaction in a stroke event, have demonstrated that, in considerable proportion of cases, the first contact of the individuals was with a primary healthcare physician, despite stating that they would call the emergency medical services in such an event.

The results of this study have identified higher level of educational attainment or having experienced a stroke previously as the independent predictor factors of higher stroke knowledge, similar to the findings of certain previous studies [25,32,57,58]; although studies with contrary findings have also been reported previously [23,34,45,59]. In regard to educational background, there may be a greater level of interest in the subject and a greater accessibility of the person to the sources of information [58,59]. In case of recurrent stroke, the information that the patients receive during admission and upon discharge is a key factor, which allows them to identify the symptoms to be compatible with a new event and to undertake the necessary actions [33,58,59,60].

Despite the privileged position of healthcare professionals in regard to improving patients’ awareness of stroke, as well as the importance of their behavior and healthy lifestyle choices [51], only a small number of people stated that doctors and nurses were the main source of their knowledge regarding stroke. Most of the patients have stated that they acquired their knowledge through personal or family experiences, or through media, television in particular. The figures obtained in this study is similar to those noted in the review studies reported by Teuschl et al. [54] and Jones et al. [56]. Healthcare professionals, therefore, are required to adopt a more active role in educating their patients regarding stroke, especially in the case of patients who are at the greatest risk of having stroke.

In view of these results, awareness campaigns are required, targeting the people who are at the greatest risk of stroke in particular, in addition to the general public, underlining the association of the symptoms with disease, their potential seriousness, the requirement for treatment of the modifiable RFs, and how avoiding delays in the visit to the hospital is related to a more positive prognosis. Briefly, simple messages which are consistent and repetitive should be used, expressed in a straightforward, direct language that is easy to remember and adapt, according to the sociocultural reality of the audience [44,54]. Certain authors have observed that although the mass-media campaigns result in immediate improvement in the knowledge, the awareness of stroke RFs and symptoms declines post intervention [61]. A review article reported by Mellon et al. [62] concluded that the studies that manage to reduce the pre-hospital delays among stroke patients exhibit a combined focus on media, targeted community education, and professional education. The awareness campaigns should start much earlier, in school (e.g., children, emphasis on the existence of therapies that can improve outcomes and minimize disability) and at the university stage (emphasis on the fact that symptoms of stroke, even when they do not seem severe, are the manifestation of a serious disease process requiring immediate medical intervention) [63].

This study had some limitations. First, only the most common stroke RFs were collected, not recording information about the prevalence of dietary or psychosocial factors in the patients. Second, this study was conducted in a tertiary care referral center for neurological disease (unicentric study), with a small sample, which makes it difficult to generalize the results. Third, the interviews were not conducted immediately after admission, which implies that the patient knowledge might be lower than those revealed in this study. The choice of some exclusion criteria (such as in-hospital stroke, level of consciousness, severe aphasia, mild patients discharged within the first hour after admission or refusal to participate in the study), may affect the outcome.

The strength of this study was the exhaustive data collection and the method used for collecting these data, using open-ended questions, which, although resulting in the underestimation of knowledge, provided a more accurate picture of the actual situation. In regard to future research in this area, the development and validation of a Spanish questionnaire for the subject are recommended about these important issues. Previously, stroke studies had focused on the stroke symptom knowledge or awareness and socio-demographic variables. This study has reported about knowledge of RFs and warning signs of stroke and how they were acquired, as well as the attitude towards a possible stroke event associated with seeking medical care. This information constitutes a fundamental pillar in the decision making process and delays within stroke patients’ route to the hospital, having a very important influence on arrival time, a critical aspect for improving patient outcome.

## 5. Conclusions

In conclusion, this study highlighted a low level of knowledge of warning signs and stroke RFs among the hospitalized patients. A previous stroke or secondary/higher education levels are the predictor factors, which increase the probability of knowledge of warning signs, RFs, or reaction to possible event. Recognizing the warning signs of stroke or knowing how to react in a possible stroke event may reduce the time delay between the event and the admission to hospital, increasing the number of patients who could be candidates to receiving fibrinolytic therapy or endovascular revascularization. Understanding the RFs of a stroke may ensure that the patient is aware of their own situation, encouraging behavioral changes and a better following of gradual treatment programs intended for the prevention of new and recurrent cases.

## Figures and Tables

**Table 1:** Socio-demographic and clinical characteristics of the sample.

Factors	*n* (%)
**Socio-demographic**	
Age: Years—X (SD)	75.39 (±12.67)
Sex: Male	295 (55.77)
Marital status: Married/Living with a partner	342 (64.65)
Educational level: No formal level/Primary level	250 (47.26)
Employment situation: Active	69 (13.04)
Mean annual income: < EUR 20,000	410 (77.50)
Living situation: Accompanied	312 (58.98)
Residence area: Urban	324 (61.25)
**Past medical history**	
High blood pressure	357 (67.49)
Diabetes mellitus	132 (24.95)
Dyslipidemia	262 (49.52)
Overweight/Obesity	343 (64.84)
Cardiovascular disease	263 (49.72)
Previous stroke	124 (23.44)
Active smoke habit	98 (18.53)
Excessive alcohol intake	241 (45.56)
Sedentary lifestyle	245 (46.31)
**Stroke symptoms**	
Loss of strength/weakness	353 (66.73)
Sensitive symptoms	70 (13.23)
Speech/language disturbances	312 (58.98)
Alteration of vision	50 (9.45)
Dizziness/instability	127 (24.01)
Headache	73 (13.80)

*n*: number of patients; X: mean; SD: standard deviation.

**Table 2:** Comparison between stroke symptoms and the patients’ ability to recognize it as such.

Stroke Symptom	Patient Recognized the Symptom as Characteristic of a Stroke		*p*-Value
	Yes—*n* (%)	No—*n* (%)	
**Loss of strength/weakness**	107 (20.23)	246 (46.50)	0.982
**Yes**	53 (10.02)	123 (23.25)	
**No**			
**Sensitive symptoms**	19 (3.59)	51 (9.64)	0.366
**Yes**	102 (19.28)	357 (67.49)	
**No**			
**Speech/language disturbances**	40 (7.56)	272 (51.42)	0.481
**Yes**	23 (4.35)	194 (36.67)	
**No**			
**Affected vision**	8 (1.51)	41 (7.75)	0.09
**Yes**	43 (8.13)	437 (82.61)	
**No**			
**Dizziness-instability**	28 (5.29)	100 (18.90)	0.474
**Yes**	75 (14.18)	326 (61.63)	
**No**			
**Headache**	16 (3.02)	57 (10.78)	0.508
**Yes**	82 (15.50)	374 (70.70)	
**No**			

*n*: number of patients.

**Table 3:** Multivariate analysis of factors related to stroke knowledge.

	Knowledge of Risk Factors	Knowledge of Warning Symptoms	Knowledge of Action to Be Taken
	**OR (95%CI)**	**OR (95%CI)**	**OR (95%CI)**
**Educational level: Secondary/Higher** **Previous stroke: Yes**	3.15 (1.75–5.67)	3.19 (1.70–5.74)	1.82 (1.16–2.86)
	4.08 (2.41–6.91)	3.54 (2.09–5.99)	2.11 (1.29–3.46)

OR: odds ratio; CI: confidence interval; OR greater than 1 indicates a positive association with knowledge of risk factors, warning signs or action to be taken.

**Table 4:** Comparison between a stroke risk factor (RF) and the patients’ ability to recognize it as such.

Risk Factors	Patient Recognized the Risk Factor as Characteristic of Stroke		*p*-Value
	Yes—*n* (%)	No—*n* (%)	
**Age**			
**≤75 years**	7 (1.32)	203 (38.37)	0.074
**>75 years**	24 (4.54)	295 (55.77)	
**Previous stroke**			
**Yes**	16 (3.02)	108 (20.42)	0.002
**No**	17 (3.21)	388 (73.35)	
**High blood pressure**			
**Yes**	81 (15.31)	275 (51.99)	0.953
**No**	39 (7.37)	134 (25.33)	
**Diabetes mellitus**			
**Yes**	14 (2.65)	118 (22.31)	0.713
**No**	39 (7.37)	358 (67.67)	
**Dyslipidemia**			
**Yes**	12 (2.27)	250 (47.26)	0.857
**No**	13 (2.46)	254 (48.01)	
**Overweight/Obesity**			
**Yes**	15 (2.84)	329 (62.19)	0.873
**No**	7 (1.32)	178 (33.65)	
**Cardiovascular disease**			
**Yes**	57 (10.77)	206 (38.94)	0.528
**No**	64 (12.10)	202 (38.19)	
**Sedentary lifestyle**			
**Yes**	13 (2.46)	232 (43.86)	0.271
**No**	23 (4.35)	261 (49.33)	
**Active smoker**			
**Yes**	39 (7.37)	59 (11.15)	<0.001
**No**	58 (10.96)	373 (70.52)	
**Excessive alcohol consumption**			
**Yes**	16 (3.02)	226 (42.72)	0.12
**No**	10 (1.89)	277 (52.37)	

*n*: number of patients.

## Supplementary Materials

The following are available online at https://www.mdpi.com/2077-0383/9/8/2557/s1, Supplementary file: STROBE Statement—Checklist of items included in reports of cross-sectional studies.
